# Head-to-head comparison of three stool calprotectin tests for home use

**DOI:** 10.1371/journal.pone.0214751

**Published:** 2019-04-18

**Authors:** Sjoukje-Marije Haisma, Anne Galaurchi, Shatha Almahwzi, Joy A. Adekanmi Balogun, Anneke C. Muller Kobold, Patrick F. van Rheenen

**Affiliations:** 1 Department of Paediatric Gastroenterology Hepatology and Nutrition, University of Groningen, University Medical Centre Groningen, Groningen, the Netherlands; 2 Department of Laboratory Medicine, University of Groningen, University Medical Centre Groningen, Groningen, the Netherlands; Holbæk Hospital, DENMARK

## Abstract

**Objective:**

Treatment decisions in inflammatory bowel diseases are increasingly based on longitudinal tracking of calprotectin results. Many hospital laboratories measure calprotectin levels in sent-in stool samples with an enzyme-linked immunosorbent assay (ELISA). Several manufacturers introduced a lateral flow–based test with software application that turns a smartphone camera into a reader for quantitative measurements. We compared three home tests (IB*Doc*, QuantonCal and CalproSmart) and companion ELISA tests (fCAL, IDK-Calprotectin and Calprotectin-ALP) to see if measurement pairs agreed sufficiently.

**Design:**

A method comparison study was conducted with stool samples from patients with active or quiescent inflammatory bowel disease. Medical students without any specific laboratory training carried out the home tests with two iOS (iPhone 6 and 7) and two Android devices (Samsung Galaxy S6 and Motorola Moto G5 Plus). Two experienced laboratory technicians measured the calprotectin concentration with the ELISA method. Primary outcome was test agreement (defined as percentage of paired measurements within predefined limits of difference). Secondary outcome included reading error rate (RER) per smartphone type.

**Results:**

We performed 1440 smartphone readings and 120 ELISA tests. In the low calprotectin range (≤500 μg/g) IB*Doc*, QuantOnCal and CalproSmart showed 87%, 82% and 76% agreement with their companion ELISAs. In the high range (>500 μg/g) the agreement was 37%, 19% and 37%, respectively. CalproSmart and QuantOnCal had significantly higher RERs than IB*Doc* (respectively 5.8% and 4.8%, versus 1.9%). Forty-three percent of reading errors was on the Motorola device, in particular with the QuantOnCal application.

**Conclusions:**

All three calprotectin home tests and companion ELISAs agreed sufficiently when concentrations are ≤500 μg/g. To minimize wrongful interpretation of calprotectin changes over time it is essential to always use the home test and companion ELISA of one and the same manufacturer. Manufacturers should explicitly evaluate and report the suitability of commonly used smartphones for quantitative calprotectin readings.

## Introduction

Treatment decisions in patients with inflammatory bowel disease (IBD) are increasingly based on longitudinal tracking of stool calprotectin test results. There are indications that calprotectin-guided escalation of therapy results in better short-term clinical and endoscopic outcomes than symptom-guided decisions alone.[1] Repeated calprotectin concentrations below 250 μg/g are considered to be a useful treatment target and are synonymous with a low risk for disease flare.[2]

Many hospital laboratories measure the calprotectin concentration in sent-in stool samples with an enzyme-linked immunosorbent assay (ELISA). It was generally believed that the protein was stable in stool samples for up to seven days, [3] but recent experiments have shown that calprotectin values gradually decline to approximately 65% of baseline levels after 6 days storage at room temperature.[4, 5] To prevent underestimation of disease activity stool samples collected at home should be refrigerated until arrival at the hospital laboratory, but this complicates transport. A more practical solution would be to measure the calprotectin concentration immediately after stool sample collection at home.

A new measurement method has recently been introduced commercially. The method itself and convenience of use are similar to those of a pregnancy test. By taking a photo of the test strip with a smartphone, the patient can perform quantitative measurements at home. We compared three of these home tests (IB*Doc*, Quanton Cal and CalproSmart) with the ELISA method of the same manufacturer (fCAL, IDK-Calprotectin and Calprotectin-ALP) to see which of the pairs has the best agreement. In addition, we evaluated the reading error rate (RER) per smartphone type.

## Methods

### Study design and pre-analytical sample handling

This method comparison study was performed at the University Medical Center Groningen (UMCG, the Netherlands). Between March and May 2018 IBD patients treated at the Department of Paediatric Gastroenterology volunteered in donating stool samples for this study. They defecated onto a stool collection sheet held above the toilet water at home, and transferred a sample of stool into a classical screw top container with a spatula. They described the stool consistency by completing the Bristol Stool Form Scale (BSFS), [6] and send both the screw top container and BSFS in a resealable biomaterial envelope to the Department of Laboratory Medicine. For the purpose of this study the patients themselves were not involved in the measurement of stool calprotectin. Upon arrival at the laboratory, the stool sample was homogenized and then aliquoted for analysis with two separate methods. Both aliquots were stored at -20°C until analysis.

### Measurement of stool calprotectin with home test

**Fig 1:**
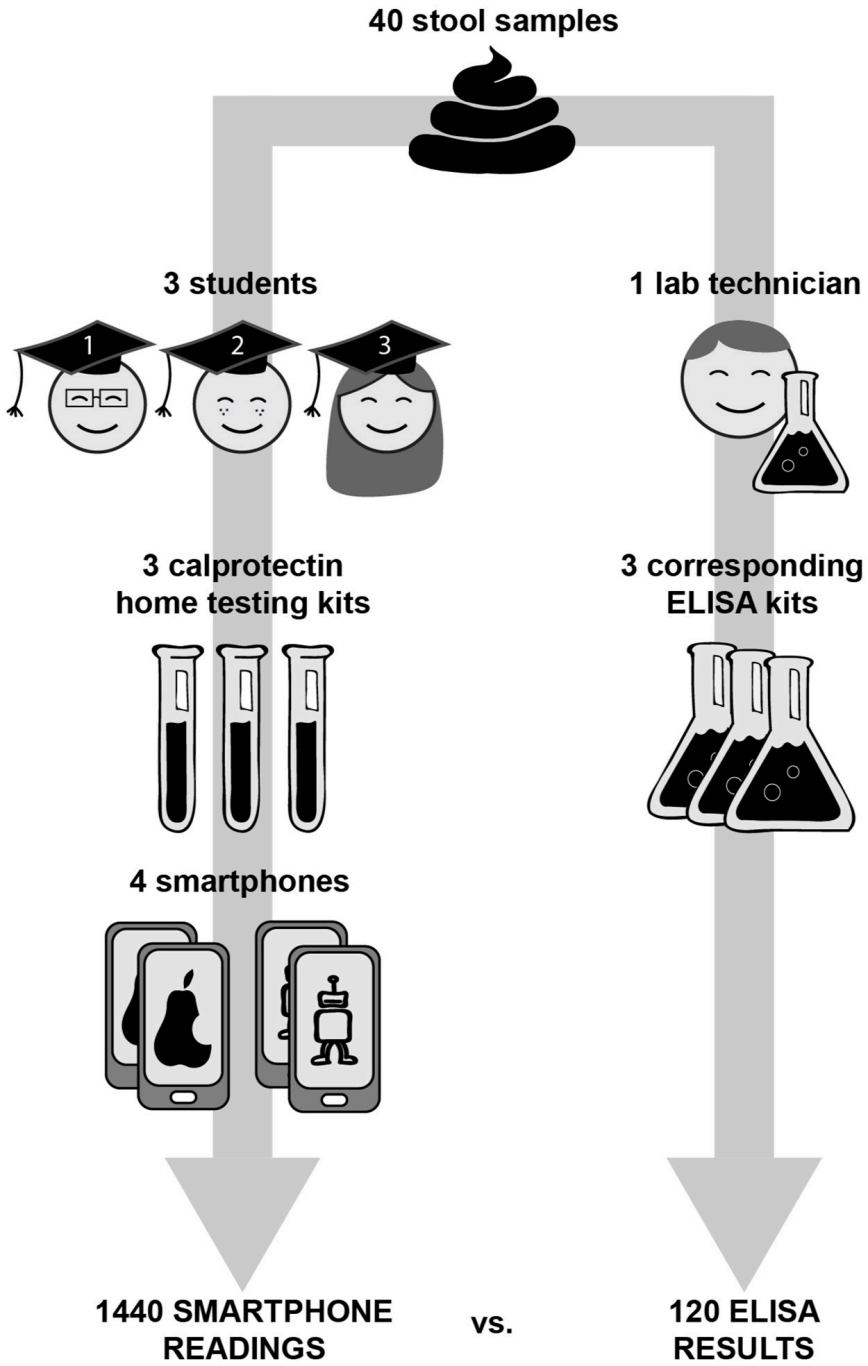
Study flow. We used 40 stool samples that covered the whole range of potential calprotectin values and performed 1440 smartphone readings and 120 ELISA measurements.

Three third year medical students without any specific laboratory training carried out the home tests of three manufacturers (Table 1). Each manufacturer-specific test cassette was read with four different smartphones which were purchased especially for this project:: two iOS (iPhone 6 and 7) and 2 Android devices (Samsung Galaxy S6 and Motorola Moto G5 Plus) (Fig 1). The sequence of use of smartphone type and home test was randomized to prevent bias. An hour before the testing session the students defrosted one of two aliquots. They all dipped the assay-specific sampling pin in the sample and transferred the fecal material into the assay-specific tube with extraction buffer. After observing the advised extract processing time, they then applied a drop of extraction fluid in the measurement window of the test cassette as per manufacturer’s instructions. After the appropriate incubation time the cassette was read with the smartphone by placing the camera above the cassette. The image was automatically analyzed by the smartphone application and the quantitative calprotectin result was directly shown on the smartphone screen.

**Table 1:** Summary of characteristics of calprotectin home tests and companion ELISAs.

	BÜHLMANN Laboratories	Immunodiagnostik AG	Calpro AG
**HOME TEST**			
App name	IB*Doc*	QuantOn Cal	CalproSmart
Extract processing time	2–24 hours	Not specified	Not specified
Incubation time test cassette	12 minutes	15 minutes	15 minutes
Test analysis time	2 minutes	2 minutes	2 minutes
Measuring range (μg/g)	30–1000 μg/g	25–2000 μg/g	70–1500 μg/g
Suitability of smartphone for single-snapshot reading of cassette			
*iPhone 6*	yes	yes	yes
*iPhone 7*	yes	yes	yes
*Motorola Moto G5 plus*	no	yes	yes
*Samsung Galaxy S6*	yes	yes	yes
**ELISA**			
Name	BÜHLMANN fCAL	IDK Calprotectin	Calprotectin (ALP)
Measuring range (μg/g)	30–1800 μg/g	25,5–2100 μg/g	25–2500 μg/g
Interassay coefficients of variation	7.8–12.8%	9.1–11.6%	4.8–13%

(derived from the manufacturer’s statements in the instruction for use).

### Measurement of stool calprotectin with ELISA

Experienced laboratory technicians, who were blinded for the smartphone readings, thawed the second aliquot and carried out batch ELISA testing with three assays that corresponded with the home tests (see Table 1). The samples were manually weighted and the measurements were done on a Dynex DS2 automated ELISA system (Alpha Labs, Easleigh, UK).

### Outcome measures

The agreement between the home test and the companion ELISA measurement was considered as the primary outcome measurement, and was analyzed with a Bland-Altman plot. As described previously [7], we reasoned that disagreement in the lower range of the tests (i.e., below 500 μg/g) could lead more easily to misclassification of disease activity than disagreement in the higher range (>500 μg/g). We therefore used predefined acceptable limits of difference, which were arbitrary set at ±100 ug/g for the lower range, and ±200 μg/g for the higher range. Secondly, concordance of home test and companion ELISA test were determined in each of 3 calprotectin ranges commonly used in our clinical practice (i.e., <250 μg/g for target range, 250–500 μg/g for undecisive range, and >500 μg/g for active disease).[7] Other outcome measures included the reading error rate (RER) per smartphone type, with reading error defined as an image of the test cassette not leading to quantitative result, and evaluation of the usability of the home test and the smartphone application on the first, seventh and last day of the home test experiments. For that purpose, the students completed the system usability scale (SUS).[8] The SUS is a simple, ten item scale giving a global view of subjective assessments of usability and learnability of a system. Scores have a range of 0 to 100, and should be considered as grade scales rather than percentages.

### Sample size calculation

The primary outcome was taken as the overall percentage of agreement between the calprotectin home test and the corresponding ELISA test per manufacturer. We wished to detect a difference in agreement of at least 15% by a two-sided test. With the level of significance set at 5% and a study power of 80%, we aimed to include at least 110 paired measurements per manufacturer.

### Statistical analysis

In Bland-Altman analysis, a scatter plot is constructed in which the difference between the paired measurements is plotted on the vertical axis and the average of the measures of two methods on the horizontal axis. The mean difference in values obtained with the two methods is called the bias and is represented by a central horizontal line on the plot. The standard deviation (SD) of differences between paired measurements is used to construct horizontal lines above and below the line to represent 95% limits of agreement (LOA). The plot enables the reader to visually assess the bias, data scatter and the relationship between magnitude of difference and size of measurement. ELISA measurements out of the measurable range of the home test were rounded to the upper limit of the home test range. In addition, we provide a distribution histogram of the differences between the paired measurements in the low range calprotectin concentrations. Concordance of home test and companion ELISA readings in each of the three calprotectin ranges are presented in a scatterplot. RERs per home test and smartphone type are reported as percentages with confidence intervals. Data were recorded electronically by using SPSS version 23.0 for Apple Mac (IBM Corporation, Armonk, NY). Graphs were constructed with GraphPad Prism version 7 for MacBook (GraphPad Software, San Diego, California USA). P-values <0.05 were considered statistically significant.

### Ethical consideration

The Medical Ethics Review Committee of the University Medical Center Groningen waived consent requirement for this specific study, as no human subjects as meant in the Dutch Medical Research Involving Human Subjects Act (WMO) were involved. Nonetheless legal guardians from all participants, as well as the children aged 12 and above, gave informed consent to use their voluntary stool samples for research. This study was conducted in compliance with the Clinical Trial Agreement, the study protocol, designated Standard Operating Procedures and the international standard for studies for In Vitro Diagnostic Medical Devices. (ISO 22870: 2016 Point-of-care testing (POCT)–Requirements for quality and competence). All authors had access to the study data and reviewed and approved the final manuscript.

## Results

Between March and May 2018, we selected 40 stool samples, of which 23 (58%) were in the low calprotectin range (<500 μg/g) and 17 (42%) in the high range (≥500 μg/g). Per homogenized stool sample 36 smartphone readings and 3 ELISA measurements were done.

### Bland-Altman analysis

**Fig 2:**
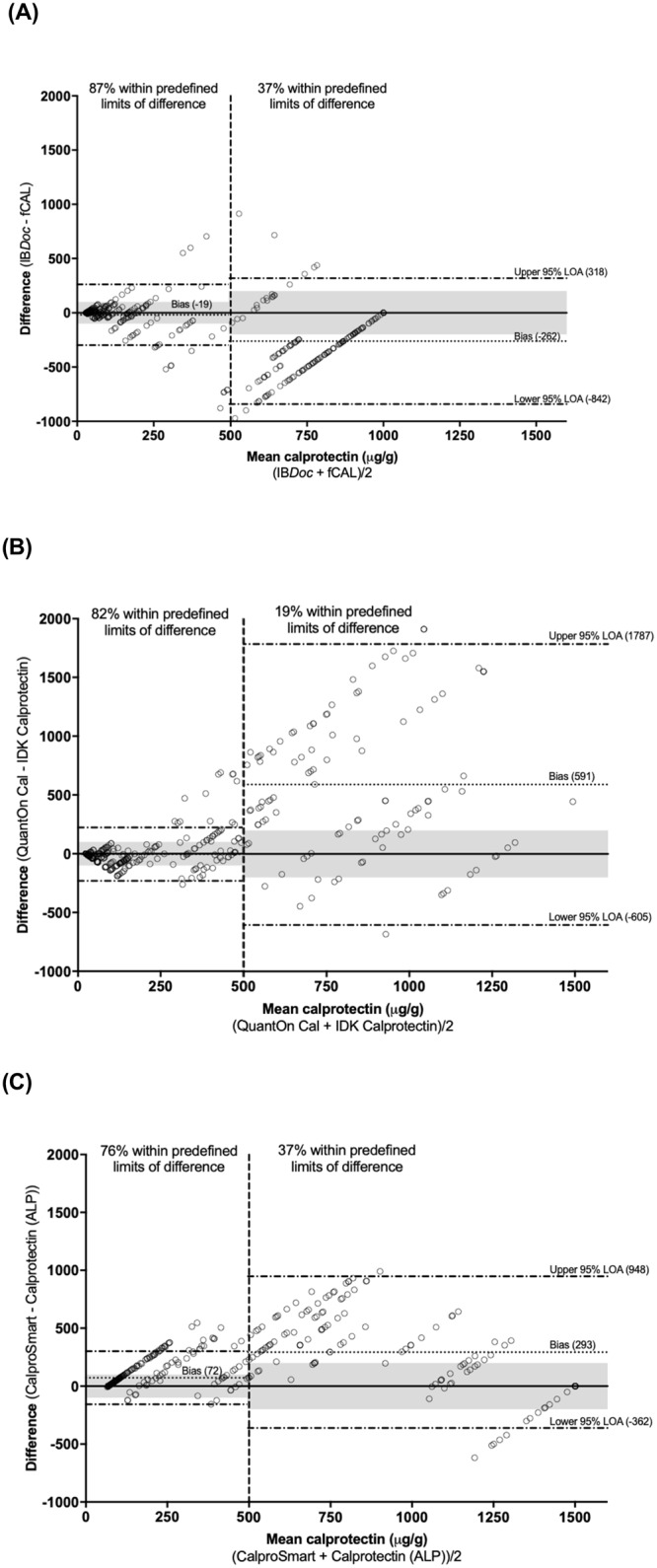
Bland-Altman plots showing difference against mean for (A) IBDoc vs. fCAL; (B) QuantOn Cal vs. IDK Calprotectin; and (C) CalproSmart vs. Calprotectin (ALP). The grey zone corresponds with the predefined acceptable limits of difference, which were arbitrary set at ±100 μg/g for the lower range and at ±200 μg/g for the higher range.[7] The dotted line is the bias (mean of the differences), the dashed lines are the upper and lower 95% limits of agreement (LOA).

In Table 2 we show the agreement between home test and corresponding ELISA per manufacturer using the Bland-Altman plot analysis. IB*Doc* underestimated the fCAL ELISA results over the whole range of potential calprotectin values with a mean bias of 105 μg/g (agreement interval 942 μg/g). QuantOn Cal overestimated IDK-Calprotectin ELISA results with a mean bias of 137 μg/g (agreement interval 1574 μg/g). CalproSmart overestimated Calprotectin-ALP ELISA results with a mean bias of 141 μg/g (agreement interval 914 μg/g). Fig 2 shows that in all comparisons the paired measurements were more clustered and closer to the zero-difference line in the low calprotectin range and more scattered in the high range.

**Table 2:** Agreement of results between three different home test and corresponding ELISA using Bland-Altman plot analysis.

	Whole range			Low range (≤ 500 μg/g)			High range (> 500 μg/g)		
	Bias (μg/g)	95% LOA (μg/g)	Agreement interval (μg/g)	Bias (μg/g)	95% LOA (μg/g)	Agreement interval (μg/g)	Bias (μg/g)	95% LOA (μg/g)	Agreement interval (μg/g)
**IB*Doc* vs BÜHLMANN fCAL**									
iPhone 7	-129	-595–337	932	-22	-295–252	548	-332	-827–164	991
iPhone 6	-87	-541–367	908	-1	-291–290	581	-246	-780–288	1067
Motorola Moto G5 Plus	-111	-617–395	1012	-41	-342–261	603	-240	-921–442	1363
Samsung Galaxy	-93	-589–363	952	-13	-270–243	513	-232	-819–356	1175
**Overall**	**-105**	**-576–366**	**942**	**-19**	**-299–261**	**560**	**-262**	**-842–318**	**1160**
**QuantOn Cal vs IDK Calprotectin**									
iPhone 7	115	-608–838	1446	-6	-255–243	498	514	-613–1640	2253
iPhone 6	169	-691–1029	1720	-12	-150–127	277	689	-509–1887	2396
Motorola Moto G5 Plus	97	-678–872	1550	-7	-257–242	499	495	-902–1893	2795
Samsung Galaxy	160	-627–947	1574	9	-247–265	512	633	-486–1752	2239
**Overall**	**137**	**-650–924**	**1574**	**-4**	**-232–223**	**455**	**591**	**-605–1787**	**2392**
**CalproSmart vs Calprotectin (ALP)**									
iPhone 7	159	-306–624	930	82	-170–333	503	322	-308–952	1259
iPhone 6	170	-294–661	955	88	-158–334	491.6	342	-337–1022	1359
Motorola Moto G5 Plus	139	-321–599	920	71	-156–298	454.4	300	-379–978	1357
Samsung Galaxy	99	-307–506	813	48	-136–232	367	212	-418–843	1260
**Overall**	**141**	**-316–598**	**914**	**72**	**-157–301**	**458**	**293**	**-362–948**	**1310**

A negative number in the “bias” column indicates that use of the home test underestimates the calprotectin concentration as compared to the ELISA method.

#### Low calprotectin range (≤ 500 μg/g)

**Fig 3:**
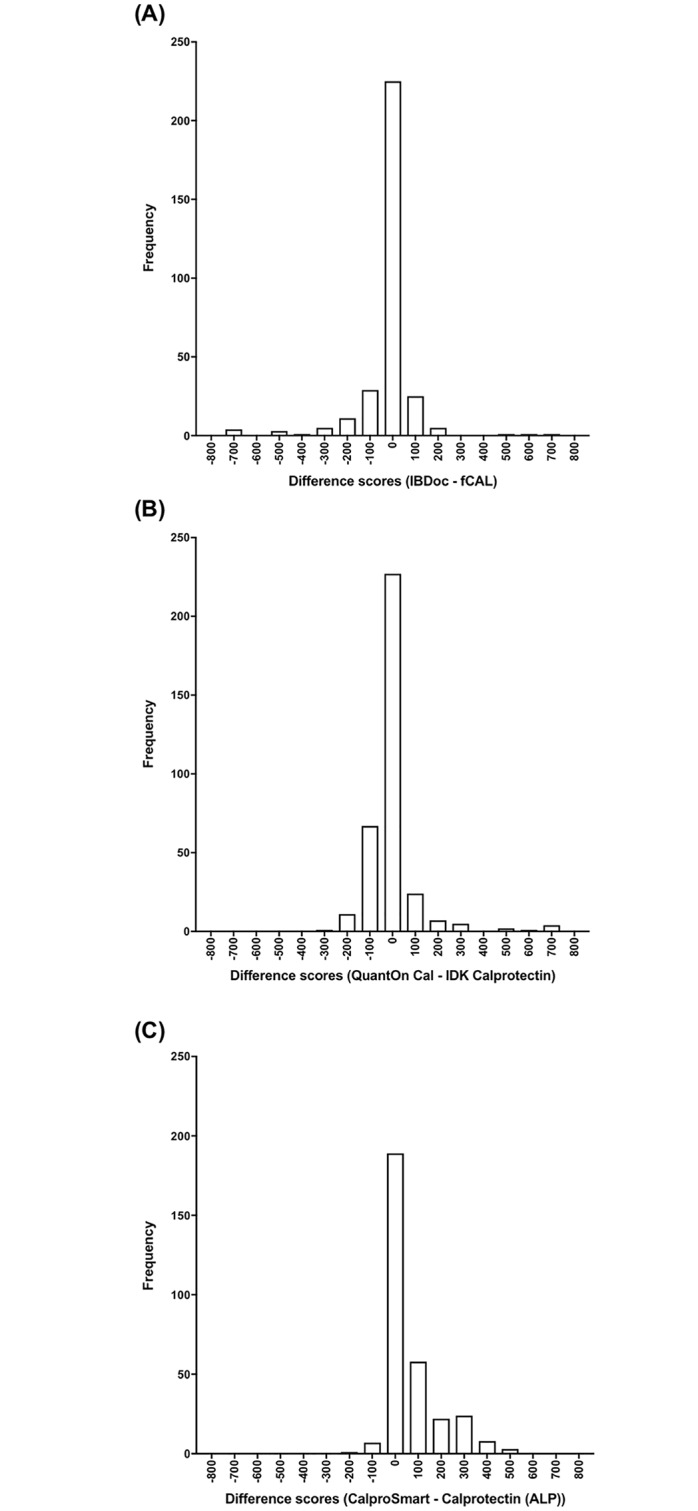
Histogram of differences in calprotectin concentrations (μg/g) for (A) IBDoc minus fCAL; (B) QuantOn Cal minus IDK Calprotectin; and (C) CalproSmart minus Calprotectin (ALP).

In the low calprotectin range the percentage of paired measurements within acceptable limits of difference for IB*Doc*, QuantOn Cal and CalproSmart were 87% (263/304), 82% (285/349) and 76% (235/310), respectively. Fig 3 shows that the difference scores for IB*Doc*, QuantOn Cal and CalproSmart were distributed in an approximately normal pattern around the bias of -19 μg/g (agreement interval 560 μg/g), -4 μg/g (agreement interval 455 μg/g) and 72 μg/g (agreement interval 458 μg/g), respectively. The agreement interval of all three comparisons exceeded the acceptable limits of difference in the low range (i.e. 200 μg/g).

#### High calprotectin range (>500 μg/g)

In the high calprotectin range the percentage of paired measurements within acceptable limits of difference for IB*Doc*, QuantOn Cal and CalproSmart were 37% (61/167), 19% (20/108) and 37% (52/142), respectively. The difference scores were not normally distributed around the bias (data not shown). The bias for IB*Doc*, QuantOn Cal and CalproSmart was -262 μg/g (agreement interval 1160 μg/g), 591 μg/g (agreement interval 2392 μg/g) and 293 μg/g (agreement interval 1310 μg/g), respectively. The agreement interval of all three comparisons exceeded the acceptable limits of difference in the high range (i.e. 400 μg/g).

### Concordance

**Fig 4:**
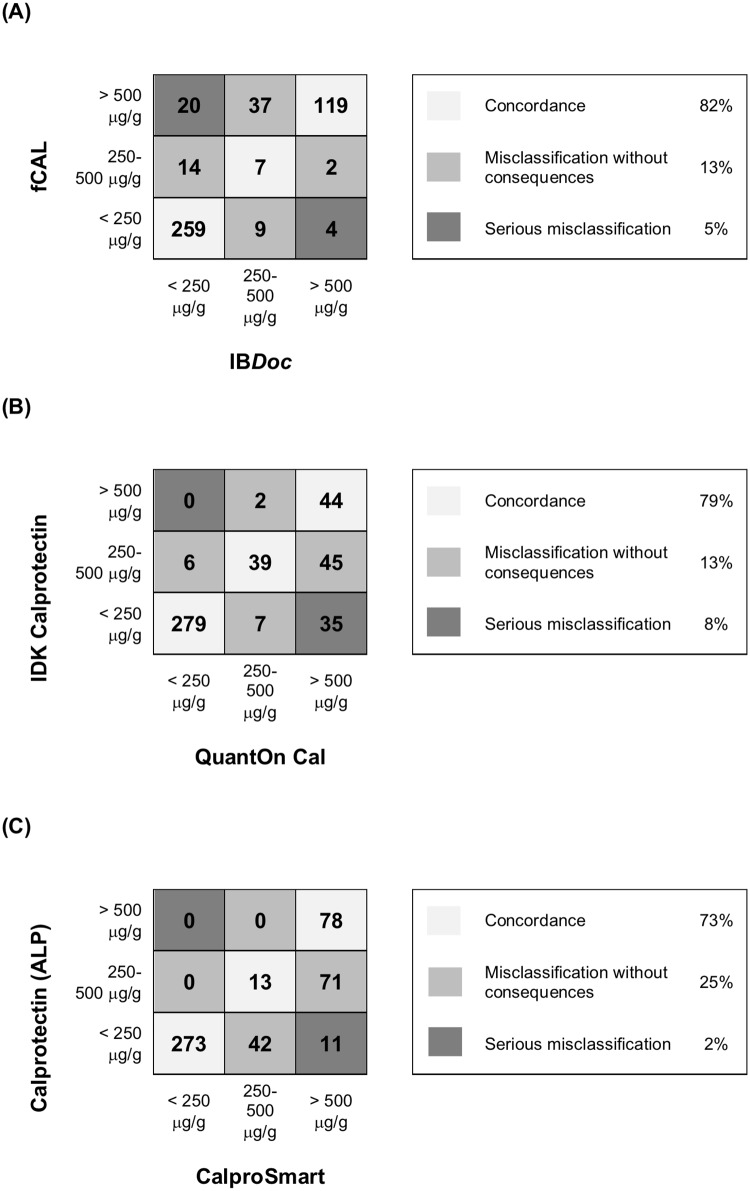
Simplified concordance matrices showing calprotectin readings with (A) IBDoc against fCAL; (B) QuantOn Cal against IDK Calprotectin; and (C) CalproSmart against Calprotectin (ALP).

Fig 4 shows the concordance between home tests and corresponding ELISA tests in each of three ranges (i.e., <250, 250–500 and >500 μg/g) that we use in our clinical practice. Of the IB*Doc*-fCAL pairs 358 of 471 (82%) were concordant. Discordance between the IB*Doc*-fCAL pairs leading to serious misclassification of disease activity (i.e., calprotectin >500 μg/g with one method and <250 μg/g with the other) were observed in 24 of 471 stool samples (5%). QuantOn Cal and IDK-Calprotectin had 361 of 457 (79%) concordant test pairs, and 35 of 457 (8%) discordant test pairs leading to serious misclassification of disease activity. CalproSmart and Calprotectin-ALP had 330 of 452 (73%) concordant test pairs, and 11 of 452 (2%) seriously discordant test pairs.

### Reading error rate

**Fig 5:**
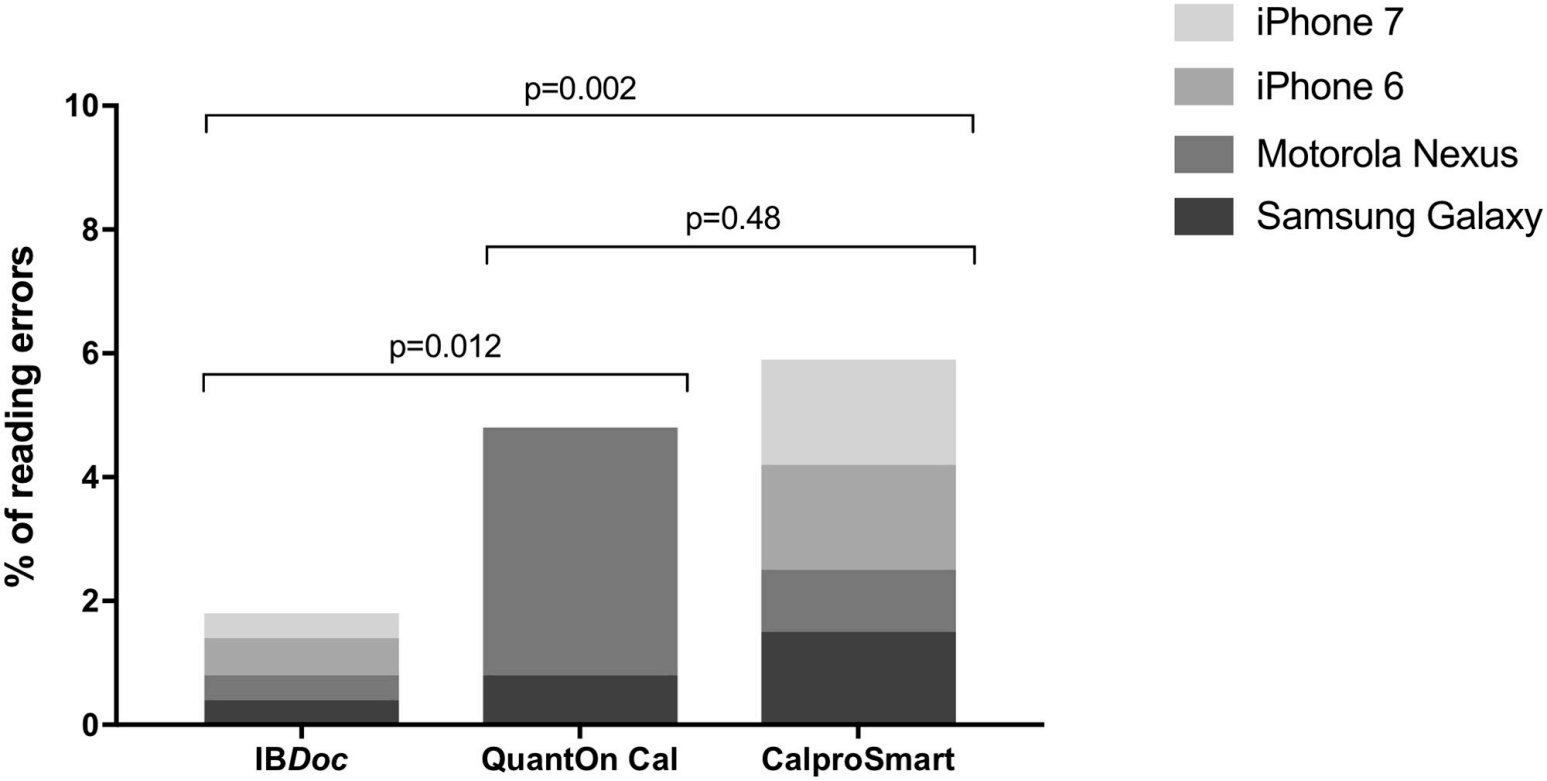
Reading error rate per home test for different smartphone types.

The CalproSmart and QuantOn Cal smartphone applications had significantly more reading errors than the IB*Doc* application, with rates of respectively 5.8% and 4.8% versus 1.9% (P = 0.002 and P = 0.012). Forty-three percent of the total amount of reading errors was with the Motorola device, in particular in combination with the QuantOn Cal application (Fig 5). Common reason for reading errors was an out of focus image.

### System usability scale

**Fig 6:**
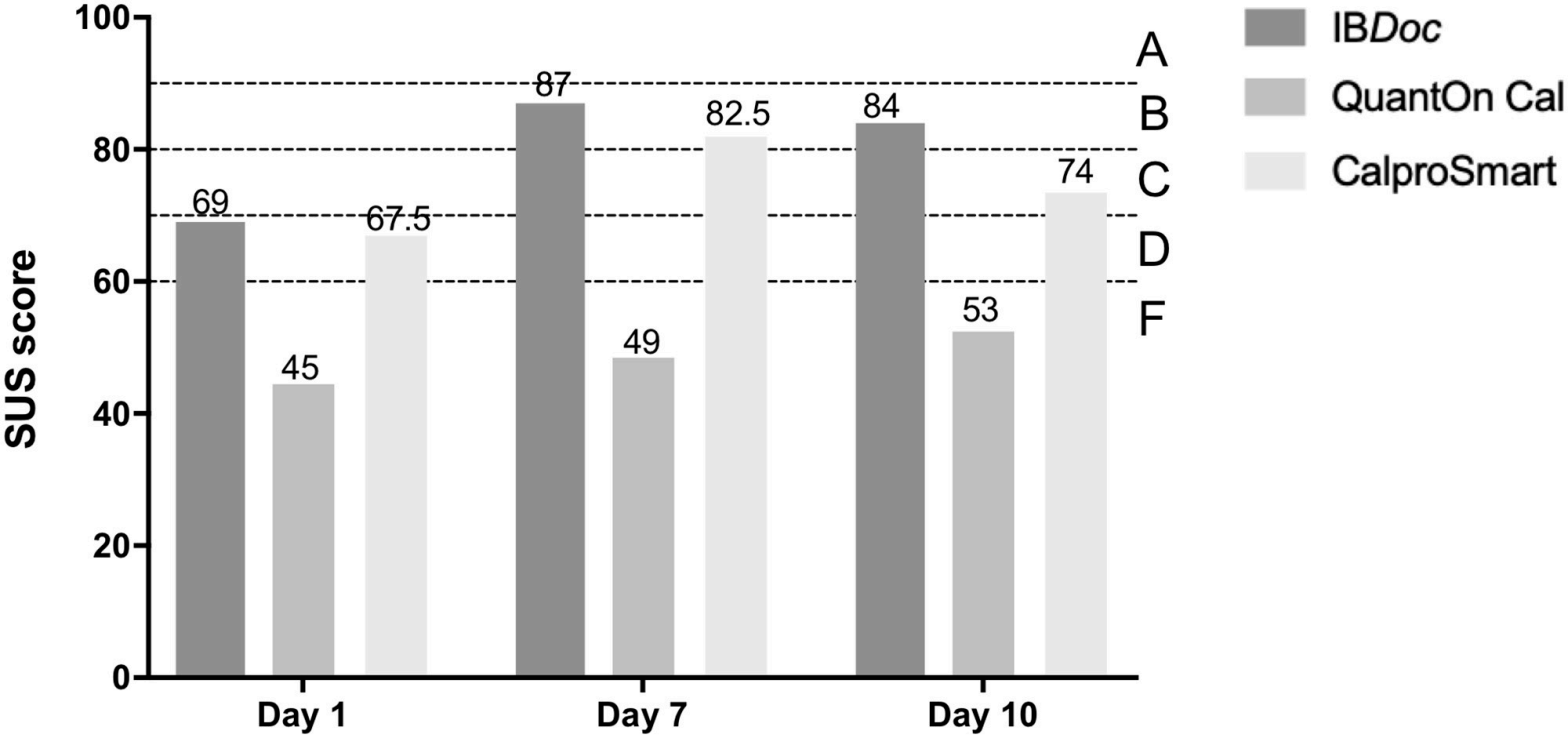
Student’s mean system usability scale (SUS) scores on day 1, 7 and day 10 of testing. SUS scores above 90 should be converted to “A”; 80–90 to “B”; 70–80 to “C”; 60–70 to “D”; and below 60 to “F”.

Fig 6 shows that mean SUS scores per home test were lowest on the first day of testing. On the last day of testing IB*Doc* was awarded the highest grade (B) of all home tests, mainly because the smartphone application was error-friendly and therefore less cumbersome to use.

## Discussion

This is the first head-to-head method comparison study investigating three calprotectin home tests and their companion ELISA tests. We have shown that the majority of measurements performed with a lateral flow immunoassay and smartphone reader agreed sufficiently with the ELISA-based quantification of the same manufacturer, provided that calprotectin levels are below 500 μg/g. In the high calprotectin range a substantial proportion of measurement pairs exceeded the predefined limits of difference (±200 μg/g). QuantOn Cal performed significantly poorer in the high range than IB*Doc* and CalproSmart. IB*Doc* had significantly fewer reading errors than the other two applications. The Motorola device turned out to be less suitable for calprotectin readings than the other smartphone types. A complete summary of the test performance per manufacturer is shown in S1 Table.

The possibility to measure calprotectin with a smartphone at home is a novelty in IBD-care. We identified three recently published studies on the IB*Doc* home test, [7, 9, 10] and one on the CalproSmart home test.[11] All studies showed acceptable agreement between home test and companion ELISA in the low range and lack of agreement in the high range. These studies differ from ours in several important ways. First, in three of the aforementioned studies, home tests were performed before shipping the unrefrigerated stool samples to the laboratory.[7, 10, 11] The possible decline in calprotectin during transport may have affected the agreement with the ELISA results, while in our experiment the transport of stool samples was taken out of the equation. Second, none of the studies performed a subgroup analysis per smartphone type, while we compared several iOS and Android devices. By doing so, we were able to show that not all smartphone cameras were suitable for calprotectin readings. Third, in this study we report an a priori decision of acceptable limits of difference for adequate interpretation of the Bland-Altman plot, a feature that was absent in three of the aforementioned studies.[9–11] Precision is of utmost importance in the low ranges of calprotectin values, where small deviations can lead to misclassification of disease activity and wrong treatment decisions. In our practice, however, imprecision in the high range is less of an issue, since we consider each calprotectin result >500 μg/g to reflect active disease, irrespective of the absolute concentration. A twofold increase in calprotectin, e.g. from 1000 to 2000 μg/g, does not necessarily mean that the inflamed surface area of the gastrointestinal tract doubled, neither that the intensity of the inflammation increased. Any shift of calprotectin values out of the target range, and into the action range (≥ 500 μg/g) is a trigger for us to change the treatment plan. In order to appreciate the true value of the home tests in the high range, we also evaluated the concordance with companion ELISAs. We observed that 119 of 125 IB*Doc* readings ≥ 500 μg/g were concordant with fCAL results (95%), compared to 35% and 49% for the QuantOnCal—IDK-Calprotectin and CalproSmart—Calprotectin-ALP pairs.

There are some limitations in our study that need to be addressed. First, the home tests were not performed by real IBD patients. Instead, we had three 3^rd^ year medical students (AG, SA, JAAB) without any specific laboratory training to fill the lateral flow cassettes and execute the smartphone readings. They only had access to the instructions for use and video tutorials on the internet. We acknowledge that their level of education is higher than the “average IBD patient”. Second, although we aimed to collect stool samples that covered the whole range of potential consistencies, we missed the liquid samples. For that reason we were unable to evaluate the effect of stool consistency on the precision of the lateral flow-based method.

The evidence base for calprotectin-guided treatment escalation and de-escalation is accumulating.[1, 2, 12] Simultaneously, the number of telemonitoring initiatives for IBD care is rising.[13–16] Telemonitoring with calprotectin home testing can make the service to IBD patients more efficient, as the technology allows to select and target patients that benefit from a face-to-face encounter with their IBD-team at short notice.

We evaluated by how much the home tests differed from the trusted ELISA method, and found that in the lower ranges the difference was small enough not to cause problems in interpretation. We conclude that the flow-based home-test and the companion ELISA method can be used interchangeably. To minimize wrongful interpretation of calprotectin changes over time it is essential to always use the home test and companion ELISA of one and the same manufacturer. This advice was already in force for the use of ELISA tests.[17–20] Ultimately, patients and health care providers would both benefit from better co-calibration of stool calprotectin assays.

## Supporting information

S1 Table Summary of performance of calprotectin home tests. (DOCX)
