# Supplementary material for: Head-to-head comparison of three stool calprotectin tests for home use
Source: PLoS One. 2019 Apr 18;14(4):e0214751. doi: 10.1371/journal.pone.0214751 (PMC6472756; doi:10.1371/journal.pone.0214751)
Supplement: S1 Table — (DOCX) [file pone.0214751.s001.docx]

|  | **Primary outcome** | | **Secondary outcomes** | | |
| --- | --- | --- | --- | --- | --- |
|  | Agreement (low range) | Agreement (high range) | Concordance | RER | SUS |
| IB*Doc* | 😐 | ☹ | ☺ | ☺ | ☺ |
| QuantOn Cal | 😐 | ☹ | 😐 | 😐 | ☹ |
| CalproSmart | 😐 | ☹ | 😐 | 😐 | 😐 |
| ☺: good; 😐: moderate; ☹: poor. | | | | | |
